# Variations in drug-related problems detected by multidisciplinary teams in Norwegian nursing homes and home nursing care

**DOI:** 10.1080/02813432.2018.1499581

**Published:** 2018-08-23

**Authors:** Siri A. Devik, Rose Mari Olsen, Inger Lise Fiskvik, Terje Halbostad, Tone Lassen, Natalia Kuzina, Ingela Enmarker

**Affiliations:** a Centre of Care Research Mid-Norway, Steinkjer, Norway;; b Faculty of Nursing and Health Sciences, Nord University, Namsos, Norway;; c Centre for Development of Institutional and Home care Services in Nord- Trøndelag, Stjørdal, Norway;; d Namsos Hospital, Hospital Pharmacy, Namsos, Norway;; e Apotek 1, Malvik, Norway;; f Department of Laboratory Medicine Children’s and women’s Health, Norwegian University of Science and Technology, Trondheim, Norway;; g Faculty of Health and Occupational Studies, University of Gävle, Gävle, Sweden

**Keywords:** Drug safety, older patients, primary healthcare, medication review, Norwegian Classification Tool for DRPs, cross-sectional study

## Abstract

**Objective:** Traditionally, nursing homes have been associated with suboptimal drug therapy and drug-related problems (DRPs). In contrast, less is known about drug safety in homecare. The aim of this study was to describe and compare DRPs in older persons across two care settings: nursing homes and home nursing care.

**Design:** Cross-sectional study using descriptive and inferential statistics.

Setting: Nursing homes (n = 5) and home nursing care units (n = 8) across nine municipalities in the middle of Norway.

**Participants:** Multidisciplinary medication reviews for 61 nursing home residents and 93 patients receiving home nursing care performed over the 2013–2014 period, were mapped and examined (*N* = 154).

**Main outcome measures:** DRPs classified by a Norwegian Classification Tool.

**Results:** In all, 740 DRPs were detected in the total sample, 227 in nursing homes and 513 in home nursing care. DRPs were significantly higher among patients receiving home-based care (Mean =5.5) compared to patients in nursing homes (Mean =3.7, *p* = 0.002). Among the problem categories, the need for additional drug was most frequent in nursing homes (*p* = 0.001), while documentation discrepancies reached the highest numbers in patients receiving home nursing care (*p* = 0.000). Additionally, patients in home nursing care had more problems concerning adverse reactions (*p* = 0.060); however, this was not statistically significant. Differences in DRP categories leading to changes in the patients’ medication lists were also discovered.

**Conclusions:** The frequency of unclear documentation and adverse reactions found in the homecare setting is alarming. This is an important issue given the trend in aged care towards caring people in their own homes. Further research is warranted to explore how different care settings may influence the safety of pharmacotherapy for older persons.

**Key Points**
Drug related problems are a significant cause of concern among patients receiving home nursing care as well as for patients living in nursing homes. The findings of this study showed that:
•Significantly more DRPs were detected among patients receiving home nursing care than patients living in nursing homes.
•While patients living in nursing homes were often undermedicated, documentation discrepancies were more frequent in home nursing care.
•DRP categories leading to changes on the medication lists differed between the settings.

## Introduction

Drug regimens among patients in primary healthcare settings are increasingly complex and potentially harmful [1]. In this context, the majority of the patients are of advanced age, have multiple chronic diseases and polypharmacy [1–3]. This combination of factors renders this population especially vulnerable to drug-related problems (DRPs), hospitalisation and death [4–6]. A DRP is defined as “an event or circumstance involving drug therapy that actually or potentially interferes with desired health outcomes” [7]. While the incidence and effects of potentially harmful medication therapy have been studied extensively in nursing home populations and hospitalised patients, data concerning DRPs in community-dwelling elderly populations is relatively scarce.

It is thought that the prevalence of inappropriate drug prescribing among nursing home residents and long-term care facilities is suggested to vary from 18.5% to 82.6% [8], and 14.7% to 28% among community-dwelling older adults [9–11]. However, direct comparison is hampered because studies apply different criteria for identifying DRPs or inappropriate prescriptions. Halvorsen et al. [12] found significant differences in the quality of medication prescribing in Norwegian home nursing services as compared with nursing homes. Their results indicated that while suboptimal drug therapy is equally as prevalent in the homecare setting as it is in nursing homes, there are differences in the nature of these discrepancies.

Norway’s increasingly aging population challenges the resources of an already overburdened healthcare system. As such, homecare services are becoming critically important, not only to cut costs, but to satisfy consumer preferences [13,14]. Patients in homecare are increasingly old and frail, consume multiple medications and transfer frequently between different care settings and therapists [13,15]. General practitioners (GPs) and nurses responsible for these patients often collaborate without physically working together and may maintain separate medication lists for patients they have in common. GPs also rely largely on nurses to make assessments and to communicate their observations [16]. Other characteristics such as a high degree of patient autonomy, problems with compliance, increased workloads and situational variables unique to each home can exert an environmental influence on patient safety in general and on medication safety in particular [16–18].

The risk of DRP is largely a factor of the number of drugs used, the presence of comorbidity and the patient’s age [1]. Notwithstanding, factors implicit in the care setting may also contribute to the incidence of DRPs; however, to the best of our knowledge, no studies have yet explored this important question. Therefore, the aim of this study was to describe and compare DRPs in older persons, identified by multidisciplinary teams, across two care settings: nursing homes and home nursing care.

## Material and methods

Data for this study was based on a cross-sectional study of DRPs identified through multidisciplinary medication reviews performed for 154 patients. The design was quantitative, and made use of descriptive and inferential statistics.

### Setting

The study population was recruited from nine municipalities representing both urban (> 5000 inhabitants) and rural areas (<5000 inhabitants) in the middle of Norway. From December 2013 to June 2014, these municipalities participated in the Norwegian Patient Safety Programme, “In Safe Hands,” which started as a Campaign and has continued as a Programme (2014–2019) [19]. Aiming to reduce DRPs and enhance drug safety among patients in nursing homes and home nursing care, the key intervention in this programme is a structured medication review. Health professionals (GPs, nurses and pharmacists) from each of the municipalities were gathered for seminars and workshops three times during the 7 months participation in the Programme. These seminars described the procedure for performing the medication reviews, based on national guidelines [20] and the Integrated Medicines Management Model (IMM-model) [21].

### Multidisciplinary medication review

The medication review comprises a package of measures that involves several steps [21].

The first measure aims to identify patients who are at risk for the development of DRPs and who have a need for a medication review. Based on the guidelines [20], the recommended criteria for review include old age, polypharmacy, significant changes in the patient’s health condition, a new diagnosis or recently prescribed a new medication, readmission to hospital or change between care levels. After the GP and the nurse have identified a patient, the nurse collects relevant patient data using a checklist focusing on prescribed medication, diagnosis, symptoms, daily functioning and physical parameters (e.g., renal status, weight, relevant blood samples). The medication review also involves taking an accurate and up-to-date inventory of all the medications the patient is taking (i.e., medication reconciliation), which means checking for discrepancies between the patient’s own list, the physician’s list and the list administered by the nursing home or homecare. The collected information is shared between the collaborating health personnel, each of them whom is charged with identifying potential DRPs and for ensuring that the treatment complies with national guidelines [22,23].

In the next step, the GP, the nurse and the pharmacist attend at a case conference during which they discuss the identified DRPs and the optimal treatment plan for the patient. The GP is responsible for the final decision on whether to act upon the identified DRPs. For example, the team may opt to cease, replace or prescribe additional medications. Alternatively, the team might simply adjust the patient’s medication dose, increase the level of monitoring required or correct documentation (these actions are reported as “acceptance” of the identified DRPs in the results section). While this process promotes multidisciplinary collaboration, [20], pharmacists are not traditionally active members of multidisciplinary teams in Norwegian nursing homes or homecare [24]. For the purpose of this study, all but one of the participating municipalities employed a pharmacist, who was collocated at either a hospital or a private pharmacy. In the one non-employing municipality, the GPs and nurses collaborated on identifying DRPs according to the guidelines given by the Patient safety Programme [20,21] without consulting a pharmacist.

### Data collection

Three pharmacists from the research group retrieved the documentation of the medication review from the medical records and used a Norwegian classification tool [23] to classify the DRPs. This tool classifies problems according to a hierarchical structure comprising six main categories (drug choice, dosing, adverse reaction, interaction, drug use and other) and 12 subcategories (see Table 2). The pharmacists were restricted to classifying DRPs already identified by the local teams and they did not perform any control or re-examination of the medication reviews.

### Statistical analysis

SPSS (v.24) for MS Windows was used for the analysis. Descriptive analysis of DRPs were performed, including frequencies, mean, standard deviation (SD), median, interquartile range (IQR), percentages, and 95% confidence intervals (CI) where appropriate. The Mann–Whitney U test, the Student’s *t*-test, and the Chi-square test were conducted to detect differences between patient groups. P-values greater than 0.05 were considered statistically significant.

## Results

### Characteristics of the sample

The sample consisted of 154 patients, 47 men and 107 women, and their ages ranged from 65 to 102 years. Patients living in nursing homes were more likely to reside in a rural municipality (*p* < 0.001), but no other significant differences were found regarding age, gender or number of regular drugs used. The median for the number of regular drugs used in the total sample was 8.0 (IQR =5.0). The minimum use was two drugs and the maximum was 16. Most medication reviews were performed by a GP, a nurse and a pharmacist in collaboration, but in 13 of the 93 cases reviewed in home nursing care, a GP and a nurse performed the reviews without a pharmacist present. Characteristics of the sample are given in Table 1.

**Table 1.** Characteristics of the sample.

Characteristics	Total sample	Nursing home	Home nursing care	*P*-value
	*N* = 154	*n* = 61	*n* = 93	
Age, median (IQR)	87.0 (9.0)	86.0 (8.0)	87.0 (11.0)	0.813^a^
Male, n (%)	47 (30.5)	17 (27.9)	30 (32.3)	0.563^b^
Urban, n (%)	50 (32.5)	5 (8.2)	45 (48.4)	0.000^b^
Number of regular drugs, median (IQR)	8.0 (5.0)	8.0 (4.0)	9.0 (5.0)	0.308^a^

a Mann–Whitney *U*

b Chi-square test.

### Drug-related problems

In total, 740 DRPs were identified in the entire sample (Mean =4.8, *SD* = 3.5). The most prevalent DRP categories identified in the total sample were “unclear documentation”^1^, and “unnecessary drug,” followed by “need for additional drug,” and “inappropriate drug choice.” Patients in home nursing care had significantly more DRPs (Mean =5.5, *SD* = 4.0) than those living in nursing homes (Mean =3.7, *SD* = 2.3). The categories “need for additional drug” and “unclear documentation” differed significantly between these groups, of which need for additional drug was most frequent in nursing homes and unclear documentation was most frequent in patients receiving home nursing care. Patients receiving home nursing care also had more problems with adverse reactions, although this was not statistically significant (See Table 2). Drugs classified in the nervous system group (ATC^2^ N-group) were most commonly involved in DRPs (psycholeptica/N05, analgesics/N02 and psychoanaleptica/N06). Drugs registered in the ATC A-group (digestion and metabolism) were the second most common (antacids/A02 most frequent). No differences were detected between the two care settings with regard to which drugs were causing DRPs.

**Table 2.** Identified and classified DRPs in nursing homes and home nursing care, per patient.

DRP Category	Total sample (*N* = 154)				Nursing home (*n* = 61)				Home nursing care (*n* = 93)				*P*-value^a^
	Count	Mean	Median	IQR	Count	Mean	Median	IQR	Count	Mean	Median	IQR	
Total number of DRP	740	4.8	4.0	4.0	227	3.7	3.0	3.0	513	5.5	4.0	4.0	0.002
Drug choice													
Need for additional drug	83	0.54	0.0	1.0	49	0.80	0.0	2.0	34	0.37	0.0	1.0	0.001
Unnecessary drug	132	0.86	1.0	1.0	63	1.03	1.0	2.0	69	0.74	0.0	1.0	0.109
Inappropriate drug choice	76	0.49	0.0	1.0	22	0.36	0.0	0.5	54	0.58	0.0	1.0	0.242
Dosing													
Too high dose	63	0.41	0.0	1.0	19	0.31	0.0	0.0	44	0.47	0.0	1.0	0.151
Too low dose	19	0.12	0.0	0.0	6	0.10	0.0	0.0	13	0.14	0.0	0.0	0.500
Sub-optimal dosing scheme	11	0.07	0.0	0.0	5	0.08	0.0	0.0	6	0.06	0.0	0.0	0.710
Sub-optimal formulation	18	0.12	0.0	0.0	11	0.18	0.0	0.0	7	0.08	0.0	0.0	0.280
Adverse reactions	33	0.21	0.0	0.0	8	0.13	0.0	0.0	25	0.27	0.0	0.5	0.060
Interactions	50	0.32	0.0	0.0	17	0.28	0.0	0.0	33	0.35	0.0	0.5	0.520
Drug use													
Inappropriate use by health personal	3	0.02	0.0	0.0	0	–	–	–	3	0.02	0.0	0.0	0.158^b^
Inappropriate use by patient	1	0.01	0.0	0.0	1	0.01	0.0	0.0	0	–	–	–	0.217^b^
Other													
Monitoring required	37	0.24	0.0	0.0	13	0.21	0.0	0.0	24	0.26	0.0	0.0	0.650
Unclear documentation	209	1.36	0.0	1.0	9	0.15	0.0	0.0	200	2.15	1.0	2.5	0.000
Not classified/complex problem	5	0.03	0.0	0.0	4	0.07	0.0	0.0	1	0.01	0.0	0.0	0.061^b^

a Student’s *t*-test for Mean

b Mann-Whitney U test for Median; IQR: Interquartile range

Within home nursing care, multidisciplinary teams consisting of a GP and a nurse identified significantly more DRPs than teams composed by a GP, a nurse and a pharmacist (Median =8.0 vs. Median =4.0, *p* < 0.013). Patients who had their medication reviewed by a GP and a nurse (*n* = 13) did not use more drugs than the rest of the sample in home nursing care. Unclear documentation was the most prevalent DRP found by teams consisting of only a GP and a nurse. Among the 13 patients, 86 documentation problems were found.

### Acceptance of problems and changed medication lists

At the medication reviews, the GPs accepted 518 of the 740 identified problems (70% acceptance) and changed their prescriptions accordingly, while 222 DRPs were rejected and led to no changes in the medication lists. In nursing homes, a 100% acceptance rate was seen according to “too low dose,” “suboptimal dosing scheme” and “inappropriate use by patient,” although these categories accounted for relatively few problems. In home nursing care, a 100% acceptance rate was seen for the categories: “suboptimal dosing scheme,” “inappropriate use by health personal,” and “not classified/complex problem,” but again, these problems were few in numbers. For a more detailed breakdown of the acceptance rate for each category, see Table 3.

**Table 3.** Proportions of accepted DRPs in nursing homes and home nursing care.

DRP Category	Total sample			Nursing home			Home nursing care		
	Detected Count	Accepted		Detected Count	Accepted		Detected Count	Accepted	
		%	95% CI		(%)	95% CI		(%)	95% CI
All categories	740	70.0	66.7–73.3	227	71.8	66.0–77.6	513	69.2	65.2–73.2
Drug choice									
Need for additional drug	83	77.1	68.1–86.1	49	79.6	68.3–90.9	34	73.5	58.7–88.3
Unnecessary drug	132	73.5	65.0–81.0	63	76.2	65.7–86.7	69	71.0	60.3–81.7
Inappropriate drug choice	76	50.0	38.8–61.2	22	50.0	29.1–70.9	54	50.0	36.7–63.3
Dosing									
Too high dose	63	66.7	55.0–78.3	19	73.7	53.9–93.5	44	63.6	49.4–77.8
Too low dose	19	78.9	60.6–99.3	6	100	–	13	69.2	44.2–94.3
Sub-optimal dosing scheme	11	100	–	5	100	–	6	100	–
Sub-optimal formulation	18	55.6	32.6–78.5	11	45.5	16.0–78.9	7	71.4	38.0–104.9
Adverse reaction	33	42.4	25.6–59.3	8	75.0	45.0–105.0	25	32.0	13.7–48.3
Interaction	50	60.0	46.4–73.6	17	82.4	64.2–100.5	33	48.4	31.4–65.5
Drug use									
Inappropriate use by health personal	3	100	–	–	–	–	3	100	–
Inappropriate use by patient	1	100	–	1	100	–	–	–	–
Other									
Monitoring required	37	56.8	40.8–97.5	13	69.2	44.2–94.3	24	50.0	30.0–70.0
Unclear documentation	209	80.9	75.5–86.2	9	33.3	2.5–64.1	200	83.0	77.8–88.2
Not classified/complex problem	5	80.0	44.9-115.1	4	75.0	32.6–117.4	1	100	–

CI = Confidence Interval.

**Figure 1.**
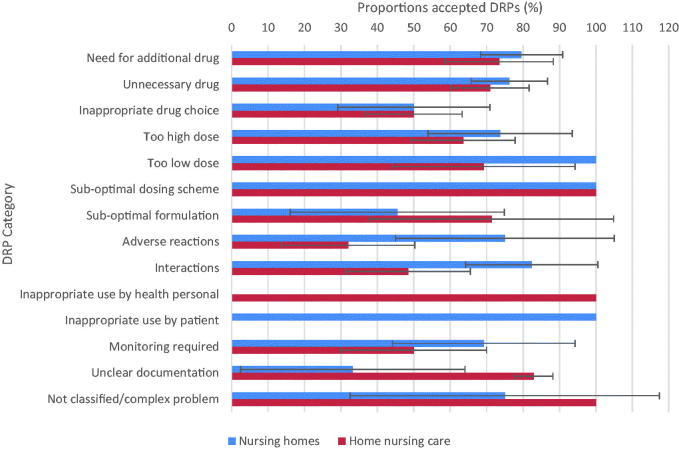
Proportions of accepted DRPs (%). Error bars represent 95% confidence intervals.

Among the categories that accounted for a high numbers of problems, physicians in both settings were most inclined to change their prescriptions according to “need for additional drug” and “unnecessary drug,” but less so for “inappropriate drug choice.” In nursing homes, the categories: “inappropriate drug choice,” “suboptimal formulation” and “unclear documentation” had the lowest acceptance rate. However, unclear documentation was rare among nursing home patients. In home nursing care, “adverse reactions,” “interaction” and “monitoring required” tended to be the least accepted DRPs (Figure 1).

## Discussion

### Main findings

Drug choice problems: “unnecessary drug,” “need for additional drug”, “inappropriate drug choice,” in addition to “unclear documentation” accounted for the majority of the DRPs found in this study. Patients receiving home nursing care had significantly more DRPs compared to patients living in nursing homes.

The categories “need for additional drug” and “unclear documentation” differed significantly between the groups. “The need for additional drug” was particularly frequent in nursing homes and “unclear documentation” was most frequent in patients receiving home nursing care.

Patients in home nursing care also had more problems regarding too high doses and adverse reactions, although neither were statistically significant. In the total sample, GPs accepted 70.0% of the DRPs and made subsequent changes to the patient’s medication regimens. The acceptance rate was 71.8% in nursing homes and 69.2% in home nursing care. Among the most frequently detected DRPs, “inappropriate drug choice” had the lowest acceptance rate in nursing homes, while ‘adverse reactions’ had the lowest acceptance rate in home nursing care.

### Strengths and limitations

Over the years, over 20 classifications systems have been developed for the identification of DRPs [24]. Having so many competing classifications systems, however, makes it difficult to compare results and to detect differences between patients and their exposure to unsafe drug treatments. We believe this study to be of particular importance given that few other studies that attempted to draw these comparisons. At the same time, there are several limitations in this study.

Data collection in this study did not include registration of the pharmacological names of all regular drugs. The number of drugs used was registered, but only those drugs identified as have caused a DRP were registered by pharmacological labels. Moreover, neither the patients’ diagnoses nor clinical conditions were included in the data. Still, the number of regular drugs used by our participants was congruent with other studies [3,5,6,8,25–30], thus suggesting that our sample had a comparable level of comorbidity.

Different GPs, nurses and pharmacists performed the medication reviews. The influence of individual professional experiences and judgements must be taken into account when considering the results. The generalisability of the results is also affected by the sampling of patients, as performed by the liable professionals at the respective units. However, the intention was to describe the findings and variations of DRPs as identified by different clinicians collaborating in teams. Obviously, a number of factors can come together and bias these findings, including the knowledge and experience of the professionals and the quality of documentation. On the other hand, objective clinicians would be restricted to identify DRPs on the basis of written information in patient records without necessarily *knowing* the patient’s personal history, preferences, earlier reactions to treatment, symptoms and daily functioning – information that, in the authors’ experiences, may be poorly documented in the records. In our opinion, the probability of identified DRPs being clinically relevant increases when local professionals perform such reviews as compared with reviews done by more detached objective clinicians. Additionally, every local team consisted of members who had received unitary training in detecting DRPs by participating in the Patient safety Programme.

Another limitation of this study was the uses of retrospective data collection. Using this approach, our data was limited only what local professionals had documented in their journals. Thus, the researchers had restricted information about the discussions that took place at the case conferences. In addition, documentation quality varied. The presence of a pharmacist in each team provided for a more standardised and structured approach to documentation as compared to teams consisting simply of GPs and nurses.

### Findings in relation to other studies

Compared to other studies using similar classification tools, the frequency of DRPs found in the total sample is consistent with studies by Leikola et al. [25] and Halvorson et al [26], although greater than what others have detected [27–31]. Of the corresponding research, only Leikola et al. [25] and Milos et al. [30] included patients from both home nursing care and assisted-living/nursing homes. Aligned with our results, Leikola et al. [25] found that DRPs were more common in the homecare setting than in assisted-living, whereas DRPs did not differ in nature across the two settings. Milos et al. [30] found no differences between the number of DRPs in community-dwelling patients and in nursing home patients.

Much like the findings of the present, comparable studies covering both healthcare settings found a high frequency of drug choice problems (e.g. unnecessary drug, inappropriate drug choice) along with dosing problems (most often dosage that was too high) [25–27,30]. However, there is partial support in the literature for disparities in terms of the type of DRPs found between patient groups. Adverse reactions and too high doses were commonly identified among patients in home nursing care [28,29], but unclear documentation is seldom highlighted as a unique DRP. As opposed to the Norwegian tool, most classification systems define unclear documentation as a potential cause of DRP and not a DRP in itself [24]. Therefore, findings related to divergence between different medication lists, incorrect, unclear or omitted information about indication, doses, dosing scheme etc. may be classified under different labels or aggregated and reflected by another overriding category. Among comparable studies, only Halvorsen et al. [26] used the Norwegian tool without modification, reporting that unclear documentation accounted for 16% of the DRPs in nursing homes. In comparison, the present study found that documentation problems accounted for 4% of DRPs in nursing homes and 39% of DRPs in home nursing care. Brody et al. [32] also stresses the frequency of medication discrepancies in the homecare setting; all 770 patients referred from hospital to homecare services had medication discrepancies, whereas 90.1% of the lists had omissions and 71% contained dosing discrepancies. Problems with unintended medication discrepancies that occur when patients transfer between different care levels or therapists are widely reported, however these are claimed to have little clinical significance [33]. Others [34] show that medication discrepancies increase the risk of hospital admissions and that older patients who lack social supports may be particularly vulnerable (e.g. older patients living alone). The problem is topical in the homecare setting where GPs and nurses often work in suboptimal systems concerning the safe and adequate exchange of information [16–18]. Incomplete medical records and errors propagating in time and systems clearly represent threats to patients’ safety as well as to professionals’ decision-making [27]. Acknowledging unclear documentation as a significant DRP may be the first step towards raising awareness around this issue and improving the quality of care.

Halvorsen et al. [12] found that drug-prescribing quality differed between patients receiving home nursing care and nursing home patients. Compared with nursing homes, patients in home nursing care used more cardiovascular drugs and fewer psychotropic drugs, and drug-to-drug interactions were identified more frequently in home nursing care. As we did not register all pharmacological names (i.e. active ingredients) for the drugs used by patients, we must be cautious when considering the prescribing quality in our sample. Notwithstanding, psychotropic drugs were involved in the majority of detected DRPs in this study, and these drugs were at least as common in the homecare settings as in the nursing homes. Drugs in this category typically increase the risk of gait instability, falls, fractures and cognitive decline in older patients [12]. Like Halvorsen et al. [12] we found a higher numbers of interactions (drug–drug) among patients in home nursing care than in nursing homes, although this difference was not statistically significant.

The proportion of identified DRPs that led to medication changes in the total sample (70.0% agreement) was higher in this study than in other studies, where agreement ranges 55%–66% [25–27,30]. This difference is most likely a product of the DRP tool itself, because if the DRP category “unclear documentation” had been excluded, the mean acceptance rate would be 58%. Additionally, the likelihood of agreement might be higher in teams consisting of local health professionals who have direct knowledge of their patients as compared to reviews done by objective clinicians.

In both settings, a low acceptance rate was seen concerning inappropriate drug choices. Moreover, the acceptance rate for adverse reactions in home nursing care was especially low. Inappropriate drugs are defined as drugs with a low benefit-to-risk-ratio, uncertain effects or that have the potential to cause adverse reactions in excess of clinical benefits [35]. Several sets of explicit quality indicators have been developed to assess the quality of prescriptions for older people, but do not seem to have reached intended goals yet [36]. Clearly, a range of factors influences the decision-making process with respect to the prescription or withdrawal of potentially inappropriate medications, including patient-oriented prioritisation, knowledge, experience and organisational characteristics of their daily practice [37]. Making patient-oriented prioritisations demands that the prescriber has adequate knowledge about patients and their situations. In home nursing care especially, the lack of contact between physicians and their patients considerably increases nurses’ responsibility to observe, assess and transfer information. Moreover, organisational characteristics in both settings can challenge the information-flow and opportunities to have interprofessional discussions. Collaboration with pharmacists is expected to increase the knowledge and awareness of doctors and nurses concerning DRPs [26]. As such, the high number of DRPs found by teams without pharmacists represents an unexpected finding. Notwithstanding, a high number of DRPs detected does not grant support for either the quality of the review or the clinical relevance of the problems being detected. On the one hand, pharmacists may lack clinical knowledge about patients’ daily functioning (knowledge possessed by GPs and nurses); on the other hand, pharmacists have pharmaceutical expertise that exceeds that of the other professionals involved in the care of such patients. Pharmacist interventions and their clinical collaboration with other healthcare professionals in the community is thought to be a powerful force for improving medication safety [25,26]. However, other studies report incongruent findings with respect to if and how the number of DRPs identified is related to the perspectives of the professionals employed to detect them [25–30].

The variations of DRPs found across the two care settings in this study, raises perhaps more questions than answers. Most importantly, the findings highlight differences, thus indicating that the homecare setting may be just as critical as nursing homes are believed to be when it comes to suboptimal medication therapy.

### Conclusions and further research

The results of this research showed that DRPs differ in both number and nature across the two care settings: nursing homes and home nursing care. The frequency by which unclear documentation and the numbers of adverse reactions were found in the homecare setting is troubling and of particular interest in light of the trend towards an increasingly aging population with older persons preferring to be cared for in their own homes. Actions to improve documentation discrepancies in the homecare setting are therefore urgent. Further research is warranted to explore how different care settings might influence drug safety among older patients.

## Notes

1 Medication discrepancy –inconsistency between two or more medication lists

2 ATC, the Anatomical Therapeutic Chemical System. WHO. http://www.who.int/classifications/atcddd/en/

## References

[1] Guthrie B, Makubate B, Hernandez-Santiago V, et al. The rising tide of polypharmacy and drug-drug interactions: population database analysis 1995–2010. BMC Med. 2015;13:74. 25889849 10.1186/s12916-015-0322-7 PMC4417329

[2] Barnett K, Mercer SW, Norbury M, et al. Epidemiology of multimorbidity and implications for health care, research, and medical education: a cross-sectional study. Lancet. 2012;380:37–43. 22579043 10.1016/S0140-6736(12)60240-2

[3] Shah BM, Hajjar ER Polypharmacy, adverse drug reactions, and geriatric syndromes. Clin Geriatr Med. 2012;28:173–186. 22500537 10.1016/j.cger.2012.01.002

[4] Beijer HJ, de Blaey CJ Hospitalisations caused by adverse drug reactions (ADR): a meta-analysis of observational studies. Pharm World Sci. 2002;24:46–54. 12061133 10.1023/a:1015570104121

[5] Gurwitz JH, Field TS, Harrold LR, et al. Incidence and preventability of adverse drug events among older persons in the ambulatory setting. JAMA. 2003;289:1107–1116. 12622580 10.1001/jama.289.9.1107

[6] Bergman Å, Olsson J, Carlsten A, et al. Evaluation of the quality of drug therapy among elderly patients in nursing homes. Scand J Prim Health Care. 2007;25:9–14. 17354153 10.1080/02813430600991980 PMC3389457

[7] PCNE on drug related problems 2009; http://www.pcne.org/working-groups/2/drug-related-problems. Accessed April 2017.

[8] Storms H, Marquet K, Aertgeerts B, et al. Prevalence of inappropriate medication use in residential long-term care facilities for the elderly: a systematic review. Eur J Gen Pract. 2017;23:69–77. 28271916 10.1080/13814788.2017.1288211 PMC5774291

[9] Leikola S, Dimitrow M, Lyles A, et al. Potential inappropriate medication use among finnish non-institutionalized people aged ≥65 years. Drugs Aging. 2011;28:227–236. 21329402 10.2165/11586890-000000000-00000

[10] Amos TB, Keith SW, Del Canale S, et al. Inappropriate prescribing in a large community-dwelling older population: a focus on prevalence and how it relates to patient and physician characteristics. J Clin Pharm Ther. 2015;40:7–13. 25271047 10.1111/jcpt.12212

[11] Galvin R, Moriarty F, Cousins G, et al. Prevalence of potentially inappropriate prescribing and prescribing omissions in older Irish adults: findings from The Irish Longitudinal Study on Ageing study (TILDA). Eur J Clin Pharmacol. 2014;70:599–606. 24493365 10.1007/s00228-014-1651-8 PMC3978378

[12] Halvorsen KH, Granas AG, Engeland A, et al. Prescribing quality for older people in Norwegian nursing homes and home nursing services using multidose dispensed drugs. Pharmacoepidemiol Drug Saf. 2012;21:929–936. 21913280 10.1002/pds.2232

[13] World health Organisation (WHO) Home care across Europe 2012. Current structure and future challenges. 2012 http://www.euro.who.int/__data/assets/pdf_file/0008/181799/e96757.pdf. Accessed February 2017.

[14] Dimitrow MS, Mykkänen SI, Leikola SNS, et al. Content validation of a tool for assessing risks for drug-related problems to be used by practical nurses caring for home-dwelling clients aged >65 years: A Delphi survey. Eur J Clin Pharmacol. 2014;70:991–1002. 24879605 10.1007/s00228-014-1699-5

[15] Hammar T, Perälä ML, Rissanen P Clients' and workers' perceptions on clients' functional ability and need for help: Home care in municipalities. Scand J Caring Sci. 2009;23:21–32. 19000091 10.1111/j.1471-6712.2007.00582.x

[16] Ellenbecker CH, Samia L, Cushman MJ, et al. Patient safety and quality: An evidence-based handbook for nurses. 2008 Rockville, MD: Agency for Health Research and Quality, U.S. Department of Health and Human Services. AHRQ Publication No.08-0043.

[17] Henriques MA, Costa M, Cabrita J Adherence and medication management by the elderly. J Clin Nurs. 2012;21:3096–3105. 22882751 10.1111/j.1365-2702.2012.04144.x

[18] Samia LW, Ellenbecker CH, Friedman DH, et al Home care nurses' experience of job stress and considerations for the work environment. Home Health Care Serv Q. 2012;31:243–265. 22974083 10.1080/01621424.2012.703903

[19] The Norwegian Patient Safety Programme 2011-2019; http://www.pasientsikkerhetsprogrammet.no/om-oss/english/the-norwegian-patient-safety-programme-in-safe-hands. Accessed February 2017.

[20] The Norwegian Directorate of Health 2012; Veileder om legemiddelgjennomganger (Guidelines for medication reviews) Avvailable from: https://helsedirektoratet.no/Lists/Publikasjoner/Attachments/465/Veileder-legemiddelgjennomgang-IS-1998.pdf.

[21] Andersen A, Wekre L, Sund J, et al. Evaluation of implemention of clinical pharmacy services in central Norway. Eur J Hosp Pharm. 2014;21:125–128.

[22] Scullin C, Scott MG, Hogg A, et al. An innovative approach to integrated medicines management. J Eval Clin Pract. 2007;13:781–788. 17824872 10.1111/j.1365-2753.2006.00753.x

[23] Ruths S, Viktil KK, Blix HS Klassifisering av legemiddelrelaterte problemer (Classification of drug related problems). Tidsskr nor Laegeforen. 2007;127:3073–3076. 18049498

[24] Basger BJ, Moles RJ, Chen TF Development of an aggregated system for classifying causes of drug-related problems. Ann Pharmacother. 2015;49:405–418. 25614526 10.1177/1060028014568008

[25] Leikola S, Virolainen J, Tuomainen L, et al. Comprehensive medication reviews for elderly patients: findings and recommendations to physicians. J Am Pharm Assoc. 2012;52:630–633. 10.1331/JAPhA.2012.10163 23023843

[26] Halvorsen KH, Ruths S, Granas AG, et al. Multidisciplinary intervention to identify and resolve drug-related problems in Norwegian nursing homes. Scand Prim Health Care. 2010;28:82–88. 10.3109/02813431003765455 PMC3442322 20429739

[27] Davidsson M, Vibe OE, Ruths S, et al. A multidisciplinary approach to improve drug therapy in nursing homes. J Multidiscip Healthc. 2011;4:9–13. 21468243 10.2147/JMDH.S15773 PMC3065561

[28] Hatah E, Tordoff J, Duffull SB, et al. Pharmacists’ performance of clinical interventions during adherence support medication reviews. Res Social Adm Pharm. 2014;10:185–194. 23688540 10.1016/j.sapharm.2013.04.008

[29] Montgomery AT, Sporrong SK, Tully MP, et al Follow-up of patients receiving a pharmaceutical care service in Sweden. J Clin Pharm Ther. 2008;33:653–662. 19138243 10.1111/j.1365-2710.2008.00965.x

[30] Milos V, Rekman E, Bondesson Å, et al. Improving the quality of pharmacotherapy in elderly primary care patients through medication reviews: a randomized controlled study. Drugs Aging. 2013;30:235–246. 23408163 10.1007/s40266-013-0057-0

[31] Fog AF, Kvalvaag G, Engedal K, et al. Drug-related problems and changes in drug utilization after medication reviews in nursing homes in Oslo, Norway. Scand J Prim Health Care. 2017;35:329–335. 29096573 10.1080/02813432.2017.1397246 PMC5730030

[32] Brody AA, Gibson B, Tresner-Kirsch D, et al. High prevalence of medication discrepancies between home health referrals and centers for medicare and medicaid services home health certification and plan of care and their potential to affect safety of vulnerable elderly adults. J Am Geriatr Soc. 2016;64:e166–e170. 27673753 10.1111/jgs.14457 PMC7293876

[33] Kwan JL, Lo L, Sampson M, et al. Medication reconciliation during transitions of care as a patient safety strategy: a systematic review. Ann Intern Med. 2013;158:397–403. 23460096 10.7326/0003-4819-158-5-201303051-00006

[34] Manias E, Annakis N, Considine J, et al. Patient-, medication-, and environment-related factors affecting medication discrepancies in older patients. Collegian 2016;24:571–577. https://www.sciencedirect.com/science/article/pii/S1322769616301445

[35] Wickop B, Langebrake C Gute Verordnungspraxis beiälteren Patienten (Good prescribing practice inthe elderly). Ther Umsch Rev Thérapeutique 2014;71:366–373. 10.1024/0040-5930/a000524 24867351

[36] Pohontsch NJ, Heser K, Löffler A, et al. General practitioners’ views on (long-term) prescription and use of problematic and potentially inappropriate medication for oldest-old patients-A qualitative interview study with GPs (CIM-TRIAD study). BMC Fam Pract. 2017;18:22. 28212616 10.1186/s12875-017-0595-3 PMC5395870

[37] Voigt K, Gottschall M, Köberlein-Neu J, et al. Why do family doctors prescribe potentially inappropriate medication to elderly patients? BMC Fam Pract. 2016;17:93. 27449802 10.1186/s12875-016-0482-3 PMC4957869

